# The roles of mutant p53 in reprogramming and inflammation in breast cancers

**DOI:** 10.1038/s41418-025-01549-w

**Published:** 2025-07-23

**Authors:** Shivaani Kummar, Marc Fellous, Arnold J. Levine

**Affiliations:** 1 Division of Hematology and Medical Oncology, Knight Cancer Institute, Oregon Health and Science University, Portland, OR USA; 2 PMV Pharmaceuticals Inc, Princeton, NJ USA; 3 The Simons Center for Systems Biology, Institute for Advanced Study, Princeton, NJ USA

**Keywords:** Tumour-suppressor proteins, Cancer genomics

## Abstract

Rezatapopt is an investigational small molecule p53 reactivator that binds specifically to the Y220C-mutant p53 protein without interacting with wild-type or other mutant p53 proteins. Upon binding, rezatapopt stabilizes the Y220C-mutant p53 protein in the wild-type conformation, reactivating p53 functions. The Phase 1 PYNNACLE trial assessed rezatapopt in solid tumors. One study participant with triple-negative breast cancer experiencing severe inflammation of the skin overlying the breast and left arm edema saw inflammation improve within 1 week of receiving rezatapopt and completely resolve shortly after. After 6 weeks of treatment, tumor volume had reduced 41%. The patient remains on study, with continued resolution of the skin inflammation and reduced tumor burden for greater than 24 months. There are several wild type Tp53 regulated pathways that could play a role in reversing the inflammatory response and tumor growth observed in this patient case. This perspective explores the signal transduction pathways involved in this cancer mediated inflammation and the extensive reduction of detectable tumor tissue.

## Introduction

The tumor suppressor protein, p53, plays many crucial roles in cellular functioning through the regulation of diverse signaling cascades [1–6]. In its role as a tumor suppressor, p53 monitors for DNA damage and cellular stress and induces apoptosis or senescence in damaged or stressed cells, preventing cancers from originating. Within normal cells, p53 regulates cellular and mitochondrial metabolism, tissue remodeling, DNA repair, and the immune response. Due to this wide range of cellular functions that the p53 protein is involved with, mutations in the *TP53* gene are associated with almost every hallmark of cancer and cancer development [7, 8]. Mutations in the *TP53* gene that encodes the full length p53 protein are a frequent occurrence in many cancers, driving tumorigenesis and cancer progression [6–8]. *TP53* mutations are estimated to occur in ~55% of all cases of human cancers, although this number can be much higher in certain tumor types [6–8].

This perspective describes a patient with triple-negative breast cancer (TNBC) and extensive inflammation of the skin overlying the left breast and left arm who responded to treatment with rezatapopt by rapidly eliminating the inflammation and shrinking the tumor volume. Rezatapopt is an investigational small molecule p53 reactivator that restores wild-type p53 function in Y220C-p53 mutated tumors [9–12]. This perspective compiles a series of observations found in the published literature that examines the role played by the loss of p53 function in driving tumorigenesis and the inflammatory responses. These inflammatory responses are seen in certain types of cancer and often involve tumor-associated epithelial and mesenchymal cells and their interactions with the components of the immune system. These observations predict that changing the mutant conformation and functions of p53 proteins, to a wild type functional p53 protein, should reduce or eliminate the inflammation and shrink the tumor tissue by one or more mechanisms of cell death [1].

## Patient case summary

Rezatapopt is an investigational first-in-class, orally bioavailable, small-molecule p53 reactivator designed to selectively bind to the p53 protein with a Y220C mutation, without interacting with the wild-type p53 protein or p53 proteins harboring other mutations [9–11]. Rezatapopt fits tightly into the pocket created by the tyrosine to cysteine substitution in the Y220C mutant p53 protein via non-covalent hydrogen bonding, which enhances hydrophobic and van der Waals interactions. Through this binding, rezatapopt selectively stabilizes the mutated protein, as shown by X-ray crystallography and nuclear magnetic resonance imaging, thereby restoring wildtype p53 protein conformation, functionality, and activity functions [9–11]. Rezatapopt is currently under investigation for the treatment of patients with locally advanced or metastatic solid tumors harboring a *TP53* Y220C mutation in the Phase 1/2 PYNNACLE clinical trial (NCT04585750) [12]. The Phase 1 portion of the PYNNACLE trial has completed enrollment, and the PYNNACLE Phase 2 registrational trial is open and enrolling. Here, we provide a case report of a patient responding to rezatapopt treatment received as part of the PYNNACLE Phase 1 study in support of the observations and the involvement of p53 loss of function in the cancerous inflammatory response discussed above.

**Fig. 1:**
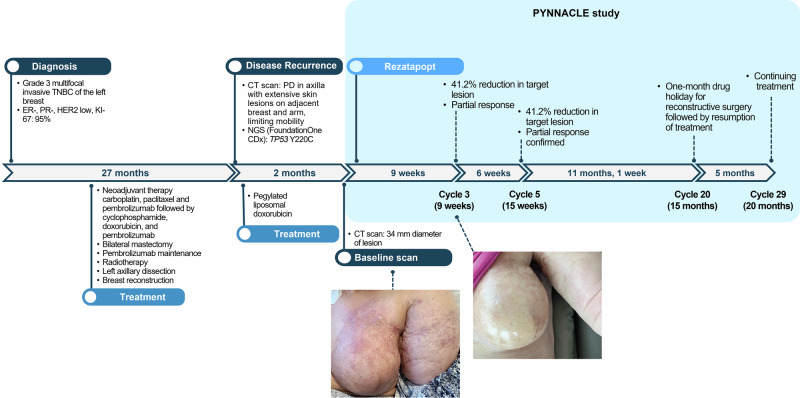
Clinical timeline and response of a patient with TNBC treated with rezatapopt in the Phase 1 PYNNACLE trial. (Upper Portion) CT computed tomography, ER estrogen receptor, HER-2 human epidermal growth factor receptor 2, PD progressive disease, PR progesterone receptor, TNBC triple-negative breast cancer.  (Lower Portion) Response of patient with TNBC to rezatapopt after 3 cycles of treatment in the Phase 1 PYNNACLE trial. (Photo Lower Left) At baseline, the patient with TNBC presented with inflammatory changes, skin breakdown, and arm edema. (Photo Lower Right) After the third treatment cycle of rezatapopt, the patient’s skin had healed with a reduction in the lymphedema of the left arm.

In December 2021, a 51-year-old woman was diagnosed with a grade 3, multifocal invasive TNBC of the left breast (Fig. 1 top). The tumor was ER and PR negative, HER-2 equivocal (+2), and FISH negative, and KI-67 was 95%. She received neoadjuvant therapy consisting of carboplatin, paclitaxel, and pembrolizumab followed by cyclophosphamide, doxorubicin, and pembrolizumab. The patient subsequently underwent a bilateral mastectomy and left axillary dissection followed by pembrolizumab maintenance therapy and radiotherapy. In March 2023, she was diagnosed with disease recurrence and developed skin nodules in the left axilla and axillary adenopathy, ulcerations of the skin overlying the left chest, and extensive lymphedema of her left arm (Fig. 1). Tumor biopsies taken from the patient and assessed using FoundationOne CDx next-generation sequencing (Foundation Medicine, Inc.) detected a *TP53* Y220C mutation. Following the diagnosis of metastatic recurrent disease, she received pegylated liposomal doxorubicin. At the time of referral for clinical trial participation, the patient presented with increasing actively replicating metastatic lesions, inflammatory changes, skin breakdown, lacerations, and significant arm edema that severely limited mobility in her dominant left arm and hand (Fig. 1 top and bottom, left).

Based on the presence of the *TP53* Y220C mutation in her tissue biopsy, the patient was enrolled in the PYNNACLE Phase 1 dose-escalation study [13]. In June 2023, she received rezatapopt 2000 mg once daily, taken with food. After 1 week of treatment, the patient’s symptoms began to improve, with rapid healing of skin ulcerations. Her skin ulcerations completely healed, and she noted improvement in edema and mobility of her left shoulder and arm (Fig. 1 bottom, right).

At 6 weeks, the patient had a partial response (41% reduction in the axilla lesion), which was confirmed at 12 weeks. This response was still ongoing as of June 2025, at the start of Cycle 29 and Month 20 of treatment, with a 1-month holiday from treatment for reconstructive surgery. At the end of the non-treatment month, the patient resumed taking rezatapopt on the original dose and schedule. The patient has tolerated rezatapopt well, with grade 2 anemia and grade 1 fatigue attributed to the study treatment. She remains on study and continues to receive rezatapopt with ongoing improvement in her left (dominant) arm and hand mobility with additional physical therapy.

Reports from the patient’s computed tomography scans show a clear response to treatment with rezatapopt. On May 31, 2023, prior to starting treatment, the patient’s scan showed ill-defined infiltrating left axillary and posterior arm enhancing soft tissue mass, with associated left subclavical vein occlusion and resultant extensive left upper extremity, chest wall, and upper abdominal soft tissue edema. On January 21, 2025, after 20 months of treatment (with a 1-month treatment holiday), computed tomography scans showed no evidence of new metastatic disease, and the treated left axillary infiltrative mass was stable and unmeasurable.

## Perspective: explanation of the role of p53 loss of function in the cancerous inflammatory responses

**Fig. 2:**
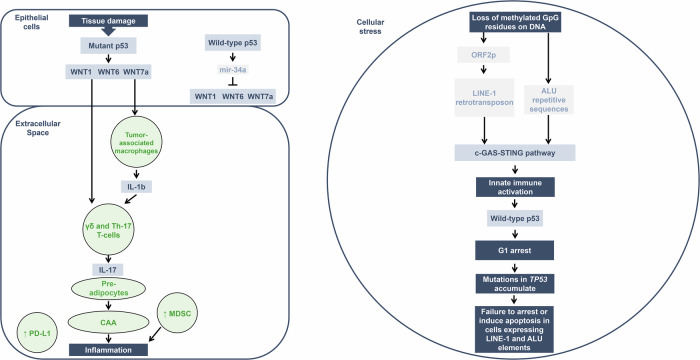
Overview of the signal transduction pathway that leads to the creation of an inflammatory cell state in triple-negative breast cancer. (LEFT) Wild-type p53 induces the transcription of mir-34a, a small non-coding RNA that negatively regulates the transcription of the *WNT* genes. The oncogene WNT is produced when both alleles of *TP53* are mutant and cell division and tissue destruction result in the induction of cytokines such as IL-1b. This leads to the accumulation of γδ and Th-17 T-cells, which secrete IL-17 and IL-1b. This tumor microenvironment influences pre-adipocytes to undergo reprogramming into CAAs and increases the number of MDSCs. Collectively, this induces an inflammatory state. Cancerous cells that have TP53 mutations can be reprogramed by demethylation CpG dinucleotides permitting the expression of Alu and LINE-1 RNA sequences, activating the gas-sting pathway resulting in interferon production. (Right side of figure). CAA cancer-associated adipocyte, IL interleukin, MDSC myeloid-derived suppressor cell.

Based on observations described in prior publications [14, 15] (Fig. 2), and the epigenetic reprogramming of cells with *TP53* mutations [16–20], there are several events and processes that can give rise to cancerous inflammatory responses (involving the WNT pathway, epithelial cells, the extracellular matrix, LINE-1, ALU repetitive DNA sequences, and cellular reactive oxygen species). What all these events and observations have in common is the loss of wild-type p53 functions caused by mutations in both alleles of the *TP53* gene, which play a critical role in initiating inflammatory responses and transcription of repetitive DNA sequences. These events ultimately give rise to interferon production triggering the immune system and genomic instability, which propagates and evolves the cancer. Here, we provide a perspective of these individual observations.

### The WNT pathway

Wild-type p53 is a transcription factor that transcribes the micro-RNA, mir-34a, which in turn negatively regulates the transcription of *WNT* genes [1, 14]. In some cancers that can be associated with inflammation, such as TNBC, mutations causing the loss of p53 function initiate transcription of *WNT* genes and WNT-dependent expression and secretion of WNT proteins (WNT-1, -6, and -7a) into the extracellular matrix as the initial step in the inflammation process often observed in these types of tumors. Tumor-associated macrophages in the matrix express WNT receptors (FZD-7 and -9), and in response to initiation of the Wnt-beta-catenin signaling pathway and the damage of breast tissue due to expanding cell numbers, interleukin (IL)-1b is synthesized and secreted into the extracellular matrix. IL-1b then engages gamma-delta (γδ) T-cells, which produce and secrete IL-17, leading to a strong inflammatory response [14].

### Epithelial cells and the extracellular matrix

Mutations occurring in both alleles of the *TP53* gene in epithelial tumor cells eliminate p53-mediated apoptosis and reprogram the cell to produce cytokines (such as IL-1b), inducing changes in the cell types found in the extracellular matrix [15]. The high levels of WNT and IL-1b attract and produce Th-17 T-cells synthesizing IL-17, a contributor to the inflammatory pathology [14, 15, 21]. In addition, loss of p53 in the stroma allows for changes in the cytokine secretion pattern that promotes myeloid-derived suppressor cells (MDSCs) that can lead to inflammatory responses influencing tumor progression [22]. The microenvironment also influences T-cell immunity in cancer and inflammation. Specifically, in breast cancer, it was shown that loss of wild-type p53 in tumor cells can lead to the compromised ability of preadipocytes to undergo terminal differentiation, and instead they were reprogrammed toward an inflammatory cell state autonomously [15]. As a result, the tumor micro-environment mediates the inflammatory T-cell response, along with MDSCs and several types of macrophages. PD-L1, an immune checkpoint protein, is also expressed on a number of these cells [14, 15].

### CpG methylation and epigenetic changes

A series of transcription factors are able to change a differentiated cell into a stem cell by reprogramming the CpG methylation sites or patterns in the genome: Oct3/4, Sox2, Klf4, and c-Myc, known collectively as the Yamanaka factors [16]. The removal of these methylated sites on CpG residues in a differentiated cell initiates p53-mediated apoptosis that lowers the efficiency of epigenetic changes and increases the time it takes to produce stem cells [17, 18]. Thus, it is wild-type p53 protein-mediated apoptosis, a response to extensive epigenetic changes, that reduces the efficiency of stem cell production and trans-determination in the presence of the Yamanaka factors. A mutant p53 protein will allow a much higher production of stem cells at a faster rate in a differentiated cell undergoing removal of CpG methylation sites [17, 18].

There is also a strong correlation between the expression of LINE-1 elements and ALU RNA sequences and the *TP53* mutation status of tumor cells [19, 20]. Epigenetic changes in the LINE-1 and ALU repetitive DNA sequences in cancer cells that have been reprogrammed because of the loss of methylated CpG residues result in the occasional expression of these repetitive elements in a cell. The LINE-1 reverse transcriptase produces DNA copies of LINE-1 mRNAs in the cytoplasm, which along with the ALU sequences, activate the c-GAS-STING pathway [20]. This pathway produces interferons that engage the innate and adaptive immune systems with the goal of eliminating the cell [20]. The ORF2p reverse transcriptase nuclease results in DNA replication intermediates and DNA breaks, which then activate wild-type p53. This results in a permanent G1 arrest where the CDKN1A gene produces a p53-inducible p21 protein [1, 20]. As this process of tumor suppression progresses, the wild-type *TP53* allele is put under a great deal of stress to function efficiently. As a result, mutations in the *TP53* gene that accumulate in cells over a lifetime will fail to induce G1 arrest or apoptosis. Therefore, mutations in both alleles of the *TP53* gene simultaneously increase the removal of the CpG methylation sites in LINE-1 elements and ALU DNA sequences of the genome and result in an inability to kill these cells [17–22]. It appears that in esophageal cells the expression of LINE-1 and Alu RNA sequences precede the Tp53 mutations, while in triple negative breast cancers the Tp53 mutations precede the expression of Alu and LINE-1 RNA sequences.

Collectively, these are reasonable hypotheses to help explain the association of chronic inflammation with a higher rate of cancers. The inflammatory response and the common role of adipocytes in this pathway could also explain why breast cancers are by far the most common cancers in patients with LFS (inherited *Tp53* mutations) and why obesity is also associated with higher rates of certain cancers [13, 23, 24].

## Discussion

Many of the observations in this article were originally made in very diverse experimental systems including cell culture and animal studies. The observations were often from different points of view, with diverse goals and interpretations, and with few or no apparent connections. Here, we describe a common set of features that relate each of these experimental observations to a patient with TNBC responding to treatment. These in vitro and in vivo observations are consistent with the roles of mutant and wild-type p53 functions in regulating inflammatory responses and cellular replication of cancers [21].

The case report presented here is only a single example that is explained well by the literature and the mechanisms of inflammation explored in this perspective. These observations reinforce the idea that the p53 protein plays an important role in the human immune system and inflammatory responses. These results strengthen the hypothesis that the wild-type p53 protein exerts a homeostatic force reducing inflammation, and the expression of mutant p53 protein is associated with a series of events (via the WNT-beta catenin pathway, IL-1b, and IL-17) that can lead to an inflammatory response. Evidently, there is a great deal of communication between the *TP53* gene functions and the immune system [13, 21–24].

These preclinical and clinical observations demonstrate a role and mechanism for how the loss of p53 regulation and function can produce inflammatory responses and continued tumor growth. Additionally, they highlight a patient case where the reversal of such inflammatory responses and tumor replication was observed with treatment in a clinical trial that restored wild-type p53 function.

## Methods

### Ethics declaration and statement of consent

The PYNNACLE Phase 1/2 study was conducted in accordance with the consensus ethical principles derived from international guidelines (Declaration of Helsinki; Council for International Organizations of Medical Sciences International Ethical Guidelines), applicable Good Clinical Practice Guidelines, and applicable laws and regulations. The study and associated documents were reviewed and approved by the Institutional Review Board/Independent Ethics Committee at each study site. Written informed consent was collected from all participants.

### ctDNA next-generation sequencing testing

A 23-gene liquid biopsy–based next-generation sequencing test was used to assess plasma samples from all patients at baseline and on treatment for the presence of *TP53* mutations. Blood samples were collected at baseline, after 3 weeks of rezatapopt treatment, and at all timepoints on study corresponding to radiographic tumor assessments.

## Data Availability

The datasets generated and/or analyzed during the current study are available from the sponsor/corresponding author on reasonable request.
